# Differential effects of dietary supplements on metabolomic profile of smokers versus non-smokers

**DOI:** 10.1186/gm313

**Published:** 2012-02-23

**Authors:** Robert C Spitale, Michelle Y Cheng, Kimberly A Chun, Emily S Gorell, Claudia A Munoz, Dale G Kern, Steve M Wood, Helen E Knaggs, Jacob Wulff, Kirk D Beebe, Anne Lynn S Chang

**Affiliations:** 1 Stanford University School of Medicine, Department of Dermatology, 450 Broadway Street, C 5334, Redwood City, CA 94603, USA; 2 NuSkin International, 75 West Center Street, Provo, UT 84601, USA; 3 Metabolon Inc., 617 Davis Drive, Durham, NC 27560, USA

## Abstract

**Background:**

Cigarette smoking is well-known to associate with accelerated skin aging as well as cardiovascular disease and lung cancer, in large part due to oxidative stress. Because metabolites are downstream of genetic variation, as well as transcriptional changes and post-translational modifications of proteins, they are the most proximal reporters of disease states or reversal of disease states.

**Methods:**

In this study, we explore the potential effects of commonly available oral supplements (containing antioxidants, vitamins and omega-3 fatty acids) on the metabolomes of smokers (n = 11) compared to non-smokers (n = 17). At baseline and after 12 weeks of supplementation, metabolomic analysis was performed on serum by liquid and gas chromatography with mass spectroscopy (LC-MS and GC-MS). Furthermore, clinical parameters of skin aging, including cutometry as assessed by three dermatologist raters blinded to subjects' age and smoking status, were measured.

**Results:**

Long-chain fatty acids, including palmitate and oleate, decreased in smokers by 0.76-fold (*P *= 0.0045) and 0.72-fold (*P *= 0.0112), respectively. These changes were not observed in non-smokers. Furthermore, age and smoking status showed increased glow (*P *= 0.004) and a decrease in fine wrinkling (*P *= 0.038). Cutometry showed an increase in skin elasticity in smokers (*P *= 0.049) but not in non-smokers. Complexion analysis software (VISIA) revealed decreases in the number of ultraviolet spots (*P *= 0.031), and cutometry showed increased elasticity (*P *= 0.05) in smokers but not non-smokers.

**Conclusions:**

Additional future work may shed light on the specific mechanisms by which long-chain fatty acids can lead to increased glow, improved elasticity measures and decreased fine wrinkling in smokers' skin. Our study provides a novel, medicine-focused application of available metabolomic technology to identify changes in sera of human subjects with oxidative stress, and suggests that oral supplementation (in particular, commonly available antioxidants, vitamins and omega-3 fatty acids) affects these individuals in a way that is unique (compared to non-smokers) on a broad level.

## Background

Cigarette smoking is well-known to associate with accelerated skin aging [1,2] as well as cardiovascular disease and lung cancer, in large part due to cellular damage from oxidative stress. Metabolites are downstream of genetic variation, transcriptional changes and post-translational modification of proteins. Hence, metabolites can most accurately capture the status of disease processes or reversal of disease processes [1]. Further, the identification of specific biomarkers has recently shed light on alterations that can occur in the metabolome due to disease and environmental changes [2,3].

The adverse health effects of cigarette smoking are well documented, and the basis of these effects is the generation of free radicals. It has been estimated that smoking a single cigarette can introduce about 10^16 ^reactive radicals into the body [4]. Cigarette smoke promotes atherosclerosis and contributes to heart disease [5,6]. In monozygotic twins where only one twin smokes, the smoker is found to have significantly worse skin damage, especially fine wrinkling [7].

Because the addictive qualities of cigarette smoking can make this habit difficult to overcome, a potential strategy to address the adverse biologic effects of smoking might be to ingest substances that might absorb the free radicals or mitigate their effects. One such category of substances comprises the oral antioxidants, taken in the form of supplements. However, the ability of oral antioxidants to be absorbed through the gastrointestinal tract, circulate through the bloodstream, and reach target organs, such as the skin or heart, is still an area of active study [8].

Recently, metabolomic profiling has emerged as a powerful tool to assess disease states, as well as the physiologic effects of drugs or environmental exposures [9,10]. For instance, metabolomic profiling has been used to associate the metabolite sarcosine with prostate cancer progression [3], defining metabolic individuality, and revealing causal effects on genotype [11].

Our current short-term study explores whether a combination of common over-the-counter antioxidants, vitamins and omega-3 fatty acids might lead to detectable changes in the serum metabolites of smokers. We explore whether these changes might be correlated with physiologic or clinically visible changes in the most visible organ of the human body: the skin.

## Materials and methods

### Study design

This study was registered with Clinicaltrials.gov as protocol #10622 and performed in accord with the Declaration of Helsinki principles.

Multiple commonly available over-the-counter antioxidant compounds (many of which are vitamins) and omega-3 fatty acids were used in the study because supplements are taken in real life as 'multivitamins', oxidative stress is known to deplete multiple antioxidants in the skin, and multiple antioxidants are more effective than a single antioxidant to combat oxidative stress [12].

Following approval by the Stanford Human Subjects' Panel, healthy female volunteers provided informed consent prior to all study procedures. Healthy female volunteers were recruited using newspaper, internet, and radio advertisements, and paper postings in the San Francisco Bay area. The arms of the study were (1) smokers and (2) non-smokers. Inclusion criteria included healthy subjects aged 50 to 70 years, Fitzpatrick skin types I or II, a body mass index (BMI) in the normal (18.5 to 24.9 kg/m^2^) or overweight (25.0 to 29.9 kg/m^2^) categories, and sun or ultraviolet exposure not exceeding 2 hours per day for one month prior to enrollment. Healthy was defined as having no uncontrolled and/or active medical problems as determined by a licensed physician. Exclusion criteria included: history of cosmetic surgery to the face (facelifts, laser procedures, and chemical peels), use of anti-aging medications (such as tretinoin) within the past 3 months prior to enrollment, use of over-the-counter anti-aging creams (excluding sunscreens) more than once per week in the month prior to enrollment, and use of dietary supplements, including vitamins, for one month prior to enrollment. In addition, a control group of non-smoking females under the age of 30 years and fulfilling all the above criteria had their blood drawn for metabolomic analysis to serve as a comparison group, but did not take any supplements.

**Figure 1:**
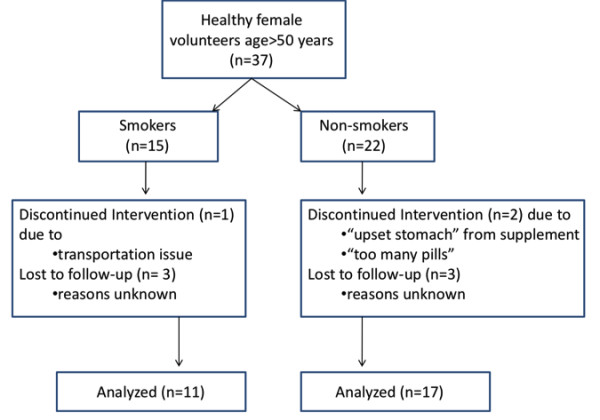
**Study flowchart**.

All subjects provided past medical histories, including smoking history and current smoking status, menopausal status, and hormone usage at baseline. Study patients were also asked to refrain from UV exposure exceeding more than 2 hours a day, including tanning bed use. In addition, they were instructed to continue their usual dietary intake and exercise, and, if they were smokers, their usual cigarette smoking, without alteration during the course of the study. Individuals who smoked at least one cigarette a day were classified as current smokers, and placed into the 'smoker' arm of the study. Subjects received a commercially available nutritional supplement containing multiple commonly available antioxidant compounds (including catechins, carotenoids, lycopene, and vitamins C, E and A), a number of common vitamins with no antioxidant properties, and omega-3 fatty acids (Additional file 1). A complete list of ingredients contained in the supplement (a commercially available product manufactured according to Good Manufacturing Practices), lifepak^® ^nano, is listed in Additional file 1 in the Product Information Page. Currently, there are no data on whether the nano size of the substances in the supplement affects bioavailability, although co-enzyme Q is five times more absorbable in nano size (NuSkin International correspondence). Study subjects were instructed to take the supplements twice daily, and were given diaries to record compliance. The study flowchart is provided in Figure 1.

Study subjects were seen for study visits at 4 weeks, 8 weeks and 12 weeks after enrollment. At each visit, adverse events and concomitant medications were ascertained. Serum was drawn at the baseline and week 12 visits only. Individuals who dropped out of the study or were lost to follow-up prior to week 12 did not have serum drawn and were not included in the study analysis. Because of diurnal variation in serum metabolites, all study procedures were conducted during a 5 hour window from 8 am to 1 pm Pacific time.

### Serum metabolite analysis

Fasting serum samples were sent for metabolomic analysis (Metabolon Inc., Durham, NC, USA) using both liquid chromatography (LC) and gas chromatography (GC) combined with mass spectroscopy (MS). The sample preparation process utilized the automated MicroLab STAR^® ^system from Hamilton Company(4970 Energy Way, Reno, NV 89502 U.S.A). Recovery standards were added prior to the first step in the extraction process for QC purposes. Sample preparation was conducted using a proprietary series of organic and aqueous extractions to remove the protein fraction while allowing maximum recovery of small molecules. The resulting extract was divided into two fractions: one for analysis by LC and one for analysis by GC. Samples were placed briefly on a TuboVap^® ^(Zymark 68A Elm Street, Hopkinton, MA 01748) to remove the organic solvent. Each sample was then frozen and dried under vacuum. Samples were then prepared for the appropriate instrument, either LC-MS or GC-MS.

The LC-MS portion of the platform was based on a Surveyor HPLC system and a linear trap quadrupole mass spectrometer (Thermo-Finnigan 81 Wyman Street, Waltham, MA 02454 USA), which consisted of an electrospray ionization source and linear ion-trap mass analyzer (Thermo Fisher Corporation 81 Wyman Street, Waltham, MA 02454 USA). The mobile phase consisted of 0.1% formic acid in H_2_O (solvent A) and 0.1% formic acid in methanol (solvent B). The extract was loaded onto an Aquasil column (100 × 2.1 mm, 3 μm, ThermoElectron Corporation 81 Wyman Street, Waltham, MA 02454 USA) via a CTC autosampler (LeapTechnologies, Carrboro, NC, USA) and gradient eluted (0% B, 4 minutes; 0 to 50% B, 2 minutes; 50 to 80% B, 5 minutes; 80 to 100% B, 1 minute; maintain 100% B, 2 minutes) directly into the mass spectrometer at a flow rate of 200 μl/minute. The linear trap quadrupole monitored positive and negative ions within a signal analysis by consecutively alternating the ionization polarity of adjacent scans.

The samples destined for GC-MS analysis were re-dried under vacuum desiccation for a minimum of 24 hours prior to being derivatized under dried nitrogen using bistrimethylsilyl-triflouroacetamide. The GC column was 20 m × 0.18 mm with a 0.18 μm film phase consisting of 5% phenyldimethyl silicone and the temperature ramp was from 40 to 300°C in a 16 minute period. Samples were analyzed on a Thermo-Finnigan Trace DSQ fast-scanning single-quadrupole mass spectrometer using electron impact ionization. The instrument was tuned and calibrated for mass resolution and mass accuracy on a daily basis.

### Metabolite identification

Biochemicals were identified by comparison to library entries of purified standards or recurrent unknown entities. The spectral files were searched using metabolomic libraries created by Metabolon Inc. that contain about 2,400 commercially available compounds. The combination of chromatographic properties and mass spectra gave an indication of a match to the specific compound or an isobaric entity. A total of 419 distinct metabolites were identified and statistically analyzed for differences between the two study arms (for a complete list of metabolites, see Additional file 2).

### Skin aging assessments

High quality digital facial photos (without makeup) with standardized lighting and 45 degree head positioning (Canfield VISIA ^®^Complexion Analysis System 253 Passaic Avenue, Fairfield, NJ 07004-2524 USA) were taken at baseline and week 12. These photos were subjected to the Canfield computerized software attached to the Complexion Analysis System to quantify physical properties of the cheek skin, including wrinkles, visible spots, UV spots, and pores. These photos were assessed by three dermatologist raters, blinded to the age and smoking status of the study subjects for clinical parameters of skin aging using a ten-point Likert scale modified from validated skin aging scales. Characteristics scored included visual softness, surface evenness, fine wrinkling, deep wrinkling, pore size, elasticity, hydration, and glow.

At baseline and week 12, physiologic measurements of facial skin barrier function and elasticity were assessed using a transepidermal water loss meter (Delphin Vapometer 62 Southfield Avenue, Suite 201, Stamford, CT 06902) and cutometer (Courage and Kazaska Cutometer^® ^MPA 580, Mathias-Brüggen-Str. 91 D-50829 Köln, Germany), respectively. The cutometer readings used for this study were mode 1, R2 = Ua/Uf. The closer the value is to 1 (100%), the more elastic the curve. To confirm intake of supplements, palmar skin carotenoid levels were measured by Raman spectrophotometry (Pharmanex Biophotonic Scanner, Provo, UT, USA).

## Statistical analysis

Statistical analysis of the metabolomic and clinical data using two-sided *t*-tests, z-tests, and ANOVA was carried out using a combination of Microsoft Excel 2007, SPSS 16.0 (SPSS Inc., Chicago, IL, USA) Oracle 10.2.0.1 Enterprise Edition, GNU R, and Array Studio (OmicSoft Corp 164 Quade Drive, Cary, NC 27513, USA). For all analyses, missing values were imputed with the observed minimum for that particular compound. The statistical analyses were performed on natural log-transformed data. Subject ratios of the 419 metabolites analyzed were calculated by dividing the subject's 12 week value by the baseline value. Similarly, ratios were calculated for the following continuous clinical parameters: VISIA^® ^assessed wrinkling, visible spots, UV spots, pore size, elasticity, and transepidermal water loss. Other clinical parameters included clinician's assessments of fine wrinkling, deep wrinkling, surface evenness, pore size, elasticity, hydration and glow.

These metabolite ratios were then compared between smokers and non-smokers by means of an analysis of covariance (ANCOVA) model, with the clinical parameter ratio being the covariate. Due to sample size, a separate ANCOVA model was fit for each clinical parameter. JMP version 8.0 was used to perform the analysis, using log transformations of both the metabolite and clinical parameter ratios. A *P*-value < 0.05 was taken to indicate statistical significance while q-values were then used to account for the false discovery rate of the identified *P*-values. For example, if a selected compound had a *P*-value of 0.035 and a q-value of 0.1, roughly 10% of all compounds with a *P*-value ≤0.035 would be a false discovery.

To get an overall view of metabolite changes in the study population at baseline and week 12, an average z-score for all the metabolites was generated for each of the two time points. The average z-score for the population was termed mScore, with a score of 0.77 indicating normal distribution. The average of the absolute values of z-scores for all metabolites at baseline and week 12 were plotted to assess deviation from normal distribution. Z-score was calculated as:

$$z=\frac{x-\mu }{\sigma }$$

where *x *is a raw score to be standardized, *μ *is the mean of the population, and *σ *is the standard deviation of the population.

To identify specific metabolites or metabolite groups with the most significant change, heat maps demonstrating fold of change over 12 weeks of each metabolite in smoker and non-smoker groups were generated, with *P*- and q-values (to account for multiple comparisons) indicating significance. Significant metabolites were searched in existing metabolomic databases (for example, the Human Metabolome Database [12], the Cofactor Database: Organic and the Wiley Online Library [13]) to cluster them into chemically or biologically related categories.

## Results

### Study patients

Fifty-eight study subjects were enrolled: 15 current smokers and 22 current non-smokers in the age category of 50 to 70 years, and 21 non-smokers in the age category of < 30 years. Four smokers and five non-smokers discontinued the study prior to the 12 week time point (Figure 1). These subjects were not included in the analysis since no subsequent serum samples were taken at a second time point after enrollment.

The average age of smokers was 56.3 ± 5.0 years and of non-smokers was 55.7 ± 6.3 years, *P *= 0.815 by *t*-test. Current smokers smoked an average of 22.8 pack-years. Body mass index, postmenopausal status, hormonal therapy and caffeine intake were not significantly different between the smoking group and non-smoking group. The basic demographics of the study subjects are shown in Table 1.

**Table 1:** Basic demographics at baseline of study subjects who completed the study

	Overall	Non-smokers	Smokers	
Characteristic	(N = 28)	(N = 17)	(N = 11)	*P*
Mean age, in years (SD)	56.0 (5.6)	55.7 (6.3)	56.3 (5.0)	0.82
Mean BMI, in kg/m^2 ^(SD)	24.7 (3.3)	24.0 (2.6)	25.5 (3.3)	0.24
Percentage postmenopausal at baseline	67.9	52.9	90.9	0.45
Percentage using hormone therapy at baseline and during study	14.3	23.5	0.0	0.19
Percentage of subjects with caffeine intake	96.4	94.1	100	0.09

The basic demographics at baseline of study subjects who completed the study were equivalent between smokers and non-smokers. *P*-values were calculated using *t*-test or Fisher's exact test (two-tailed). BMI, body mass index; SD, standard deviation.

Study supplement usage was confirmed by increases in subjects' palmar skin carotenoid levels, as measured by Raman spectroscopy. In smokers, counts indicating carotenoid levels increased an average of 15,055.25 ± 11,674.18 photon units at week 12; in non-smokers, levels increased an average of 16,640.59 ± 13,595.16 units at week 12.

### Metabolomic profiles

We identified and analyzed 419 metabolites, which consisted of known metabolites as well as unknowns.

**Figure 2:**
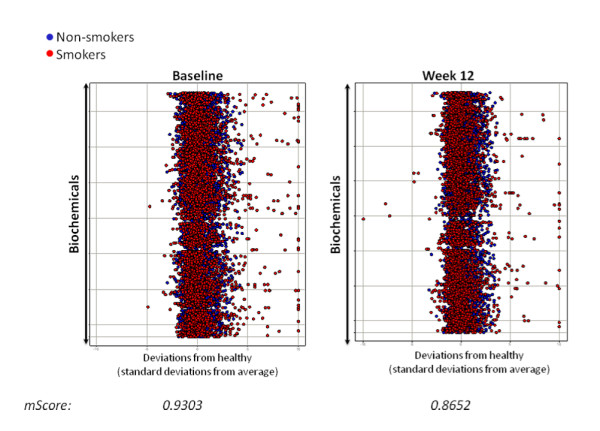
**Decreased deviation of metabolites from normal distribution after 12 weeks of study supplement, most notably in the smoker group**. Scatterplots showing absolute values of z-scores for all metabolites at baseline and after 12 weeks of taking the study supplement. The mScore is an average of all z-scores in the entire population. An mScore of 0.77 indicates a normal distribution. Red spot = smokers; blue spot = non-smokers.

To evaluate the overall effect of the study supplement on the study population, a z-score for each of the metabolites was calculated at each baseline and week 12 (Figure 2). The average of the absolute values of z-scores (termed mScore) for all metabolites at baseline and week 12 were plotted to assess deviation from normal distribution, with a normal distribution mScore equal to 0.77. At baseline, the mScore of the population was 0.930, with a significant number of smokers' metabolites (red dots) deviating from the non-smokers, or 'healthy' group (blue dots). At week 12 after supplementation, the mScore was 0.865, with supplemented smokers' metabolites becoming closer to the metabolomic profiles of the non-smoking group.

**Figure 3:**
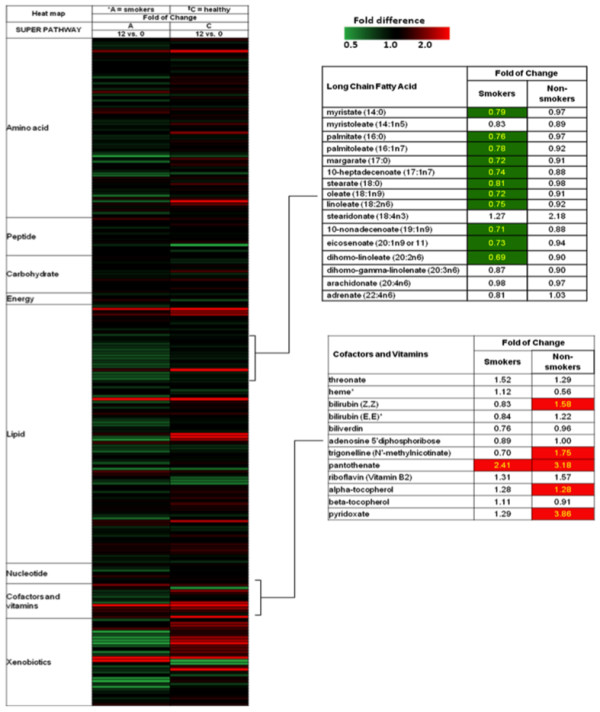
**Heat maps demonstrating fold change of metabolites in smokers and non-smokers**. The heat maps show that smokers had significantly decreased levels of (long chain fatty acids) but not the cofactors and vitamins listed below, after 12 weeks of study supplementation. Green band = decrease; red = increase

The most significant changes in metabolites between week 12 and baseline are depicted as heat maps (Figure 3), with green banding indicating decreases in metabolite (fold change < 1), and red banding indicating increases in metabolite (fold change > 1). Overall, the non-smoking group (termed the 'healthy group') displayed increases in metabolites after 12 weeks, while the supplemented smokers displayed decreases in metabolite levels (Figure 2, left).

Compared to non-smokers, the smokers at baseline had elevated levels of free fatty acids, which is consistent with previously published research [13]. The most prominent metabolomic changes after 12 weeks of study supplement ingestion was a significant decrease in most of the long chain fatty acids (LCFAs) in supplemented smokers (listed on the right side of Figure 3). In the supplemented smoking group, 11 out of the 16 LCFAs (including palmitate, stearate, oleate, and linoleate) significantly decreased. None of these 16 LCFAs changed significantly in the non-smoking group. This suggests that supplementation increasingly normalized the serum lipid levels of LCFAs in smokers. Stearidonate was the only LCFA that did not decrease over 12 weeks in the smoker group. In both smokers and non-smoker groups, stearidonate increased over 12 weeks. Of note, stearidonate is an omega-3-fatty acid, a component of the supplement. This may explain increases in stearidonate in both smoker and non-smoker groups.

Cofactors and vitamins that significantly increased after 12 weeks of supplementation are listed in the bottom right hand box of Figure 3 (shaded). Pantothenate, alpha-tocopherol and pyridoxate were significantly increased in non-smokers, as would be expected after supplementation. Interestingly, the degree of increase in smokers for alpha-tocopherol and pyridoxate was not significant, suggesting possible increased conversion, excretion or decreased absorption of these substances compared to non-smokers. Another significant change was an increase in bilirubin levels (part of the heme degradation pathway) in non-smokers after supplementation, a change not seen in supplemented smokers. The clinical and biologic significance of this change is currently unclear, although it is possible that the supplemented non-smokers increased heme breakdown or decreased excretion of bilirubin compared to supplemented smokers.

### Skin aging assessments

**Figure 4:**
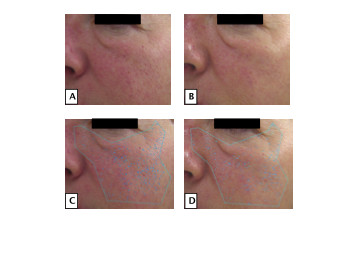
**Patient skin glow and UV spots**. **(a, b) **Example of an increase in glow at week 12 (b) compared to week 0 (a) as determined by a panel of four blinded dermatologists. **(c, d) **Example of a decrease in UV spots at week 12 (Figure d) compared to week 0 (c) in a participant as determined by VISIA Complexion Analysis System.

Smokers and non-smokers responded differently to study supplementation for 12 weeks as measured by quantitative skin aging parameters (Table 2). Smokers showed a decrease in the number of UV spots (*P *= 0.03) but increased number of pores (*P *= 0.03) on VISIA complexion analysis. On cutometry, there was a significant increase in elasticity (*P *= 0.05) in smokers. In contrast, non-smokers' values for these parameters did not change (Figure 4).

**Table 2:** Differences in facial skin aging parameters (by VISIA Complexion Analysis software), elasticity and transepidermal water loss after 12 weeks of study supplementation

Skin aging parameter	Overall (N = 28)			Non-smokers (N = 17)			Smokers (N = 11)		
	
	Mean	SD	*P*	Mean	SD	*P*	Mean	SD	*P*
Wrinkling	+0.036	14.046	0.99	-4.882	11.050	0.09	+6.615	14.239	0.12
Visible spots	+2.393	7.440	0.10	+1.235	7.471	0.51	+3.154	7.414	0.15
UV spots	-4.536	30.426	0.44	+5.000	28.588	0.48	-17.692	26.196	0.03
Pores	+2.857	9.099	0.11	-0.235	7.496	0.90	+6.385	9.341	0.03
Elasticity	+0.081	0.276	0.13	+0.020	0.274	0.77	+0.160	0.270	0.05
Transepidermal water loss (g/m^2 ^h)	-0.807	6.674	0.53	-0.941	5.459	0.487	-0.600	8.515	0.82

Smokers showed a significant decrease in UV spots and improvement in elasticity by cutometry measurement. However, smokers also displayed increased pore count. Values reported are in percentiles, except for UV spots, which are reported as absolute values. Higher percentiles represent an improvement in a parameter. Positive numbers indicate increase, negative numbers indicate decrease. SD, standard deviation.

In addition, clinical skin aging parameters that changed after 12 weeks of study supplementation (as assessed by dermatologist raters blinded to smoking status and chronological age) were not the same between smokers and non-smokers. Supplemented smokers showed decreased fine wrinkles (*P *= 0.04) and increased glow (*P *= 0.00) but decreased hydration (*P *= 0.04). Supplemented non-smokers showed decreased deep wrinkling (*P *= 0.00) and increased elasticity (0.01) (Table 3).

**Table 3:** Differences in clinical skin aging parameters after 12 weeks of study supplementation as assessed by dermatologist raters

Clinical skin aging Parameter	Overall (N = 28)			Non-smokers (N = 17)			Smokers (N = 11)		
	
	Mean	SD	*P*	Mean	SD	*P*	Mean	SD	*P*
Fine wrinkling	-0.571	2.516	0.24	+0.235	1.821	0.60	-1.769	2.743	0.04
Deep wrinkling	+0.033	3.054	0.95	-1.853	1.579	0.00	+2.500	2.754	0.01
Surface evenness	+0.429	1.874	0.24	+0.882	1.996	0.09	+0.154	1.819	0.77
Elasticity	+0.161	2.415	0.73	+1.353	1.730	0.01	-1.115	2.468	0.13
Glow	+1.179	3.151	0.06	+0.235	3.073	0.76	+2.538	2.570	0.00
Hydration	-0.786	2.234	0.07	+0.294	0.985	0.24	-1.846	2.824	0.04

Smokers showed decreased fine wrinkles and increased glow, but decreased hydration. Non-smokers showed decreased deep wrinkling and increased elasticity. Values are the average of scores given by the four raters. Negative values indicate decrease, positive values indicate increase. *P*-values calculated using two-sided *t*-tests. SD, standard deviation.

### Adverse events

No serious adverse events occurred during the course of our study. Adverse events that were deemed not related to study supplement were headaches (14), body aches (joint or muscle aches; 6) and common cold (5). Adverse events deemed possibly related to study supplement were loose stools (4), belching (4), and stomach upset (1). When these adverse events were stratified by smoker and non-smoker, none of the adverse events were significantly different between the two groups. For instance, of the 14 headaches, 3 episodes were in smokers and 11 were in non-smokers, giving a *P *= 0.313 (Fisher's exact test, two-tailed). For body aches, three episodes were in smokers and three were in non-smokers, *P *= 0.672.

## Discussion

Limitations of this study include a relatively small sample size and its short-term nature (12 weeks only). In addition, strict activity diaries kept by subjects might better record confounders such as daily sun exposure during the study.

Changes in the metabolomic profile and clinical changes observed are associations and not necessarily causative. It has been reported that increases in free fatty acids induce oxidative stress by generating reactive oxygen species and inflammation by increasing levels of NF-kB [14-16]. Since high levels of fatty acids both promote and indicate a high level of oxidative stress and inflammation, the antioxidants in the supplement could alleviate these stressors. Future studies into histologic or molecular changes might better characterize the precise changes occurring in the skin. While we are able to detect particular orally ingested antioxidants such as carotenoids in the skin, quantitative measures of other antioxidants (such as catechins) in the skin can be difficult. Measurable increased levels of these antioxidants in the skin would bolster the contention that orally ingested supplements reach the skin and can effect change.

Non-smokers in our study experienced fewer overall changes in metabolite levels, but an increase in metabolites within the vitamin/cofactor pathways. One explanation for this observation is that, upon antioxidant and vitamin supplementation, non-smokers may have had a surplus in vitamins and cofactors that remained unconsumed. Previous studies have shown that cigarette smoke directly depletes the levels of circulating antioxidants and vitamins, such as ascorbate, alpha-tocopherol, beta-carotene, lycopene, and retinol [17,18]. When taking these supplements, therefore, antioxidants and vitamins might be consumed in smokers, but left as excess in non-smokers. This supports a model in which smokers would benefit more from supplements than non-smokers.

We acknowledge that the number of cigarettes smoked per day may affect the metabolites present [19]. The total pack-years smoked varied widely in our study population: the amount ranged from 4.2 to 37.5 pack-years. Although there were variations in the amount of pack-years, we instructed the participants to not change their habits, especially the amount they smoked. From the metabolomic analysis, the levels of cotinine, a well-known metabolite of nicotine [20], did not significantly change in either group after 12 weeks of supplementation. This demonstrates that the smokers did not have drastic changes in smoking habit, suggesting that the observations we observed may have been related to the concentration of free radical oxygen species entering the circulatory system from supplementation. Further studies in which the amount of smoking are controlled could explore whether a minimum amount of smoking is required to detect significant metabolomic changes as well as define maximum levels of smoking beyond which supplementation might have limited benefit.

The decrease in the mScore of the study population toward a normal distribution (especially in the supplemented smokers) and the alteration of metabolomic heat maps in supplemented smokers suggests that the study supplement may be able to reduce the amount of metabolites generated in pathways affected by smoking. Smokers displayed more changes in their metabolome than non-smokers after study supplementation, most notably significant decreases in their LCFAs.

Of note, cigarette smoking not only introduces free radicals that promote atherosclerosis and increase the risk of cardiovascular disease [5,6], it has also been linked to changes in lipid metabolism and increased levels of free fatty acids in blood [13,21,22]. An additional explanation for the change in fatty acid composition with study supplementation is that peripheral lipolysis is attenuated and possibly accompanied by improved mitochondrial function. Lipolysis is a hallmark of insulin resistance, and has been shown to be elevated in smokers [23,24]. Any compounds within the study supplement that affect insulin sensitivity could result in decreased lipolysis and decreases in levels of LCFAs, as detected in this study. Also, components in the supplement that might improve mitochondrial function could allow for fatty acids and amino acids to be consumed more efficiently. It is possibly a combination of improved insulin sensitivity and mitochondrial function that causes the decline in fatty acids and amino acids. Evidence that supports this idea is that two markers of excessive carbon flow into the tricarboxylic acid cycle, beta-hydroxybutyrate and 2-hydroxybutyrate, decline with supplementation. These arise when either too much carbon is produced relative to tricarboxylic acid cycle capacity or the tricarboxylic acid cycle is not operating at full capacity. It also can be seen that these markers changed (only significantly for beta-hydroxybutyrate) for the non-smokers as well. Overall, these observations would suggest that the supplement improves energy metabolism for both groups but that the non-supplemented smokers simply have a more pronounced issue of higher levels of peripheral lipolysis and the fatty acids that accompany this. These observations suggest a direct link between smoking and the fatty acid composition of our sample set.

Further studies will be required to identify the specific components of oral supplementation contributing to the metabolomic changes seen in this study. In addition, further studies will need to delineate whether the decreases in LCFAs are beneficial or detrimental to overall health status. In our current study, for instance, smokers' skin improved with respect to elasticity measurements and clinical appearance of fine wrinkles, glow and hydration but displayed worsened deep wrinkling appearance after 12 weeks of study supplementation. It has been observed previously that increased fatty acid content in the skin regulates the healing process through cell-surface interactions [25]. In addition, longer-chained fatty acids have garnered a significant amount of attention for their role in skin health, which has revealed that animal models with deficient essential fatty acids experienced increased water loss through the skin [26]. These results suggest that fatty acids are a critical component of skin cell morphology and health and further supports a link between our metabolic observations and our clinical observations.

While smoking can be regarded as a 'disease condition' worthy of possible 'treatment' with oral supplementation, it is unclear whether healthy individuals such as non-smokers would benefit from oral supplementation. In fact, there are large epidemiologic studies suggesting that over-supplementation of particular nutrients can be detrimental to health [27-29]; hence, further exploration into the metabolic alterations that occur due to supplementation and their role in organ phenotypes is needed.

## Conclusions

Overall, our current pilot study suggests that metabolomic changes in cigarette smokers can be altered through oral supplementation and that these effects are different between smokers and non-smokers. Clearly, the overall and specific health consequences of these metabolomic changes need further exploration and are beyond the scope of this current study. Nevertheless, our study suggests that metabolomics can be a meaningful tool to assess the complex effects of oxidative stress in human subjects, and that it is possible to correlate clinically significant end-organ changes such as skin aging parameters with metabolomic changes. Characterization of the nature of these connections merits further study.

## Abbreviations

ANCOVA: analysis of covariance; GC: gas chromatography; LC: liquid chromatography; LCFA: long chain fatty acid; MS: mass spectroscopy; UV: ultraviolet.

## Competing interests

The authors declare that they have no competing interests. The study was performed using a research award from NuSkin International.

## Authors' contributions

ALSC conceived the project and wrote the manuscript. JW and KDB collected MS data. All authors contributed to the experiments. RCS and MYC wrote the manuscript. All authors have read and approved this manuscript for publication.

## Supplementary Material

Additional file 1 **Table S1 - heat map of statistically significant biochemicals profiled in this study**. Shaded cells indicate *P *≤ 0.05 (red indicates that the mean values are significantly higher for that comparison; green values significantly lower). Note the statistical comparison legend: *Group A = smokers; ^†^group C = healthy. Click here for file

Additional file 2 **Table S2 - adverse events during study and fold change of metabolomic profiles of smoking group after 12 weeks of antioxidant supplementation**. Click here for file
