# Supplementary material for: Differential effects of dietary supplements on metabolomic profile of smokers versus non-smokers
Source: Genome Med. 2012 Feb 23;4(2):14. doi: 10.1186/gm313 (PMC3392760; doi:10.1186/gm313)
Supplement: Additional file 2 — Table S2 - adverse events during study and fold change of metabolomic profiles of smoking group after 12 weeks of antioxidant supplementation. [file gm313-S2.DOCX]

**Table S2. A.** Adverse events during study, by order of frequency.

| **Adverse Events** | **Total Number** |
| --- | --- |
| Headache | 14 |
| Body Aches | 6 |
| Common Cold | 5 |
| Belching | 4 |
| Loose Stools | 4 |
| Dry Skin or rash | 3 |
| Heartburn | 3 |
| Itching | 3 |
| Localized Pain (Not Arthritic) | 3 |
| Abrasion | 2 |
| Arthritis | 2 |
| Bloating | 2 |
| Diarrhea | 2 |
| Eczema | 2 |
| Gas | 2 |
| Menstrual Cramps | 2 |
| Pills stuck in throat | 2 |
| Stomach Upset | 2 |
| One occurrence of the following : acne, anxiety, blushing, carpal tunnel symptom, cold sore, constipation, contusion, dry mouth, hot flash, insomnia, vaginal bleeding, nausea, psoriasis, pulled muscle, stomach flu, seasonal allergy, sinus infection, urinary tract infection, unpleasant taste | 1 |

**Table S2. B.** Fold of change of metabolomic profiles of smoking group after 12 weeks of antioxidant supplementation. P value indicates significance and q value indicates probability that the significant result is obtained by chance alone. Green cells= decrease; red cells= increase.

| **Pathway** | **Metabolites** | **Change** | **p** | **q** |
| --- | --- | --- | --- | --- |
| Glycine, serine, threonine metabolism | serine | 0.84 | 0.0071 | 0.2146 |
| Phe and Tyr metabolism | 3-(4-hydroxyphenyl)lactate | 0.79 | 0.0374 | 0.3181 |
| Phe and Tyr metabolism | 3-phenylpropionate (hydrocinnamate) | 1.20 | 0.0337 | 0.3130 |
| Val, Ile, Leu metabolism | 3-methyl-2-oxobutyrate | 0.86 | 0.0395 | 0.3181 |
| Val, Ile, Leu metabolism | 3-methyl-2-oxovalerate | 0.86 | 0.0188 | 0.2651 |
| Val, Ile, Leu metabolism | 4-methyl-2-oxopentanoate | 0.81 | 0.0090 | 0.2146 |
| Val, Ile, Leu metabolism | alpha-hydroxyisovalerate | 0.52 | 0.0036 | 0.2146 |
| Val, Ile, Leu metabolism | 2-methylbutyroylcarnitine | 0.66 | 0.0109 | 0.2146 |
| Cys, Met, SAM, Taurine metabolism | methionine | 0.90 | 0.0444 | 0.3312 |
| Cys, Met, SAM, Taurine metabolism | 2-hydroxybutyrate (AHB) | 0.72 | 0.0109 | 0.2146 |
| Arginine metabolism | assymetric dimethylarginine (ADMA) | 0.60 | 0.0220 | 0.2663 |
| Arg and Pro metabolism | N-acetylornithine | 0.64 | 0.0315 | 0.3080 |
| Butanoate metabolism | 2-aminobutyrate | 0.78 | 0.0323 | 0.3080 |
| Essential fatty acid | eicosapentaenoate (EPA; 20:5n3) | 1.84 | 0.0379 | 0.3181 |
| Medium chain FA | 10-undecenoate (11:1n1) | 0.78 | 0.0087 | 0.2146 |
| Long chain FA | myristate (14:0) | 0.79 | 0.0320 | 0.3080 |
| Long chain FA | palmitate (16:0) | 0.76 | 0.0045 | 0.2146 |
| Long chain FA | palmitoleate (16:1n7) | 0.78 | 0.0445 | 0.3312 |
| Long chain FA | margarate (17:0) | 0.72 | 0.0122 | 0.2160 |
| Long chain FA | 10-heptadecenoate (17:1n7) | 0.74 | 0.0110 | 0.2146 |
| Long chain FA | stearate (18:0) | 0.81 | 0.0125 | 0.2160 |
| Long chain FA | oleate (18:1n9) | 0.72 | 0.0112 | 0.2146 |
| Long chain FA | linoleate (18:2n6) | 0.75 | 0.0262 | 0.2709 |
| Long chain FA | 10-nonadecenoate (19:1n9) | 0.71 | 0.0147 | 0.2323 |
| Long chain FA | eicosenoate (20:1n9 or 11) | 0.73 | 0.0197 | 0.2651 |
| Long chain FA | dihomo-linoleate (20:2n6) | 0.69 | 0.0255 | 0.2709 |
| Fatty acid, dicarboxylate | 3-carboxy-4-methyl-5-propyl-2-furanpropanoate (CMPF) | 4.00 | 0.0075 | 0.2146 |
| bile acid metabolism | taurocholenate sulfate | 0.70 | 0.0241 | 0.2709 |
| ketone bodies | 3-hydroxybutyrate (BHBA) | 0.61 | 0.0214 | 0.2663 |
| Sterol/steroid | pregnendiol disulfate | 0.87 | 0.0018 | 0.2146 |
| purine metabolism | urate | 0.97 | 0.0228 | 0.2664 |
| Cofactors/vitamins | pantothenate | 2.41 | 0.0075 | 0.2146 |
| xenobiotics | iminodiacetate (IDA) | 1.34 | 0.0037 | 0.2146 |

**Table S2. C.** Fold of change of metabolomic profiles of nonsmoking group after 12 weeks of antioxidant supplementation. P value indicates significance and q value indicates probability that the significant result is obtained by chance alone. Green cells= decrease; red cells= increase.

| **Pathway** | **Metabolite** | **Fold of Change** | **p** | **q** |
| --- | --- | --- | --- | --- |
| Glycine, serine, threonine metabolism | glycine | 1.24 | 0.0026 | 0.0378 |
| Phe and Tyr metabolism | phenylalanine | 1.16 | 0.0147 | 0.1084 |
| Trp metabolism | indoleacetate | 1.31 | 0.0077 | 0.0723 |
| Val, Ile, Leu metabolism | alpha-hydroxyisovalerate | 0.75 | < 0.001 | 0.0114 |
| Val, Ile, Leu metabolism | isobutyrylcarnitine | 1.36 | 0.0069 | 0.0683 |
| gamma-glutamyl | gamma-glutamylvaline | 1.16 | 0.0141 | 0.1084 |
| glycolysis, gluconeogenesis, pyruvate metabolism | glycerate | 1.28 | 0.0174 | 0.1182 |
| Essential FA | eicosapentaenoate (EPA; 20:5n3) | 3.16 | < 0.001 | 0.0011 |
| Essential FA | docosahexaenoate (DHA; 22:6n3) | 1.68 | 0.0020 | 0.0300 |
| FA, monohydroxy | 2-hydroxystearate | 0.79 | 0.0150 | 0.1084 |
| FA, dicarboxylate | 3-carboxy-4-methyl-5-propyl-2-furanpropanoate (CMPF) | 4.04 | < 0.001 | 0.0004 |
| bile acid metabolism | glycocholenate sulfate* | 0.83 | < 0.001 | 0.0092 |
| ketone bodies | 3-hydroxybutyrate (BHBA) | 0.65 | 0.0213 | 0.1244 |
| lysolipid | 1-linoleoylglycerophospho-ethanolamine* | 0.70 | 0.0275 | 0.1349 |
| lysolipid | 1-arachidonoylglycerophospho-ethanolamine* | 0.65 | 0.0183 | 0.1182 |
| lysolipid | 2-arachidonoylglycerophospho-ethanolamine* | 0.63 | 0.0154 | 0.1084 |
| lysolipid | 2-stearoylglycerophospho-choline* | 1.34 | 0.0422 | 0.1760 |
| lysolipid | 1-docosahexaenoyl-glycerophosphocholine* | 1.75 | < 0.001 | 0.0149 |
| sterol/steroid | pregnen-diol disulfate* | 0.94 | 0.0208 | 0.1244 |
| Hb, porphyrin metabolism | bilirubin (Z,Z) | 1.58 | 0.0291 | 0.1349 |
| cofactors/vitamins | trigonelline (N'-methylnicotinate) | 1.75 | 0.0043 | 0.0527 |
| cofactors/vitamins | pantothenate | 3.18 | < 0.001 | 0.0004 |
| cofactors/vitamins | alpha-tocopherol | 1.28 | 0.0346 | 0.1545 |
| Vitamin B6 metabolism | pyridoxate | 3.86 | < 0.001 | 0.0008 |
| benzoate metabolism | catechol sulfate | 1.59 | 0.0205 | 0.1244 |
| xenobiotics | iminodiacetate (IDA) | 1.31 | < 0.001 | 0.0021 |
| sugar, sugar sub, starch | erythritol | 1.16 | 0.0291 | 0.1349 |
